# Supplementary material for: Sex difference in BAT thermogenesis depends on PGC-1α–mediated phospholipid synthesis in mice
Source: Nat Commun. 2025 Jul 14;16:6072. doi: 10.1038/s41467-025-61219-w (PMC12259864; doi:10.1038/s41467-025-61219-w)
Supplement: Supplementary file 1 — Supplementary Information [file 41467_2025_61219_MOESM1_ESM.pdf]

Supplementary Figure 1

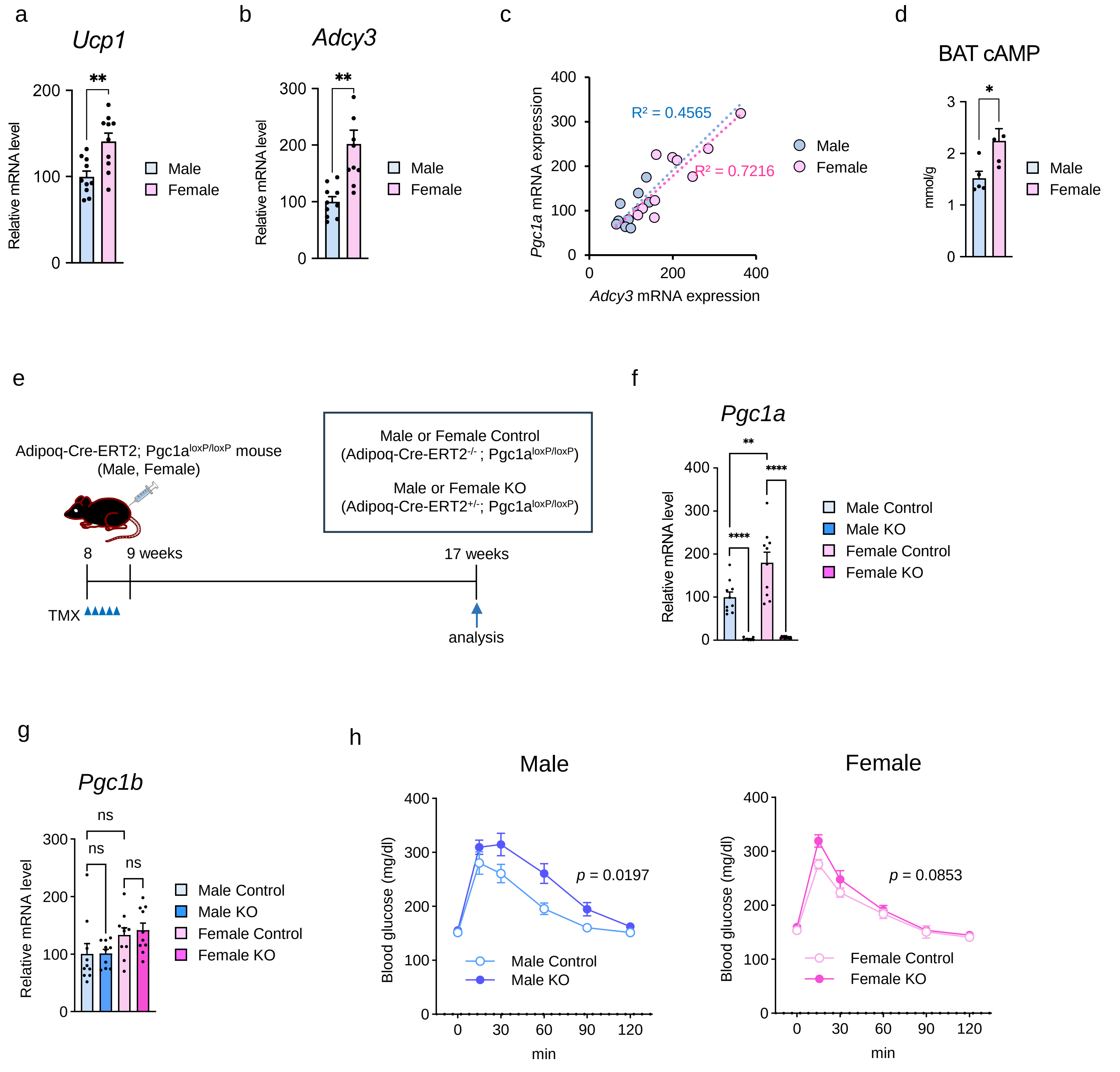

Supplementary Figure legends

Supplementary Figure 1

Thermogenesis related genes are highly expressed in BAT of female mice

(a) *Ucp1* mRNA levels in BAT of wild-type mice. n = 10 per group. $p = 0.0034$. **(b)** *Adcy3* mRNA levels in BAT of wild-type mice. n = 10 per group. $p = 0.0010$. **(c)** Correlation plots between *Adcy3* and *Pgc1a* mRNA levels in BAT of wild-type mice. n = 10 per group. **(d)** Concentration of cAMP in BAT of wild-type mice. n = 5 per group. $p = 0.0264$. **(e)** Experimental protocols of adipocyte-specific PGC-1α conditional knockout mice using tamoxifen (TMX)-inducible *Adipoq*-Cre-ERT2 mice. **(f)(g)** *Pgc1a* mRNA levels **(f)** and *Pgc1b* mRNA levels **(g)** in the BAT of Control and KO mice. n = 10 per group. $p < 0.0001$ (Male Control vs. Male KO), $p = 0.0011$ (Male Control vs. Female Control), and $p < 0.0001$ (Female Control vs. Female KO) for **(f)**. **(h)** Blood glucose levels of Control and KO mice under glucose tolerance test. n = 9–10 per group. Data are expressed as the mean ± SEM. Data were analyzed by unpaired two-sided t-test **(a, b, d)**, linear regression **(c)**, one-way ANOVA with Tukey's post hoc test **(f, g)**, and two-way repeated measures ANOVA with Bonferroni's post hoc test **(h)**. Significance is indicated (*$p < 0.05$; **$p < 0.01$; ****$p < 0.0001$). Source data are provided as a Source Data file.

Supplementary Figure 2

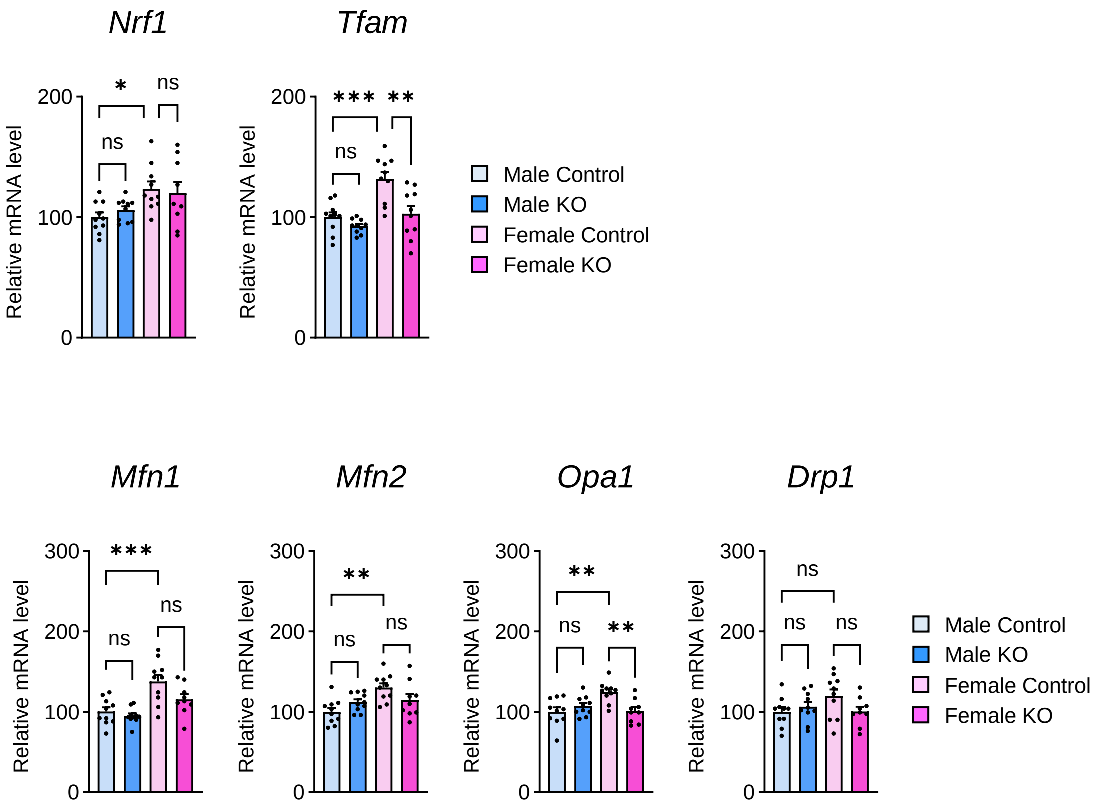

Supplementary Figure 2

Effects of PGC-1α KO on the expression of mitochondrial regulatory genes

Gene expression of *Nrf1*, *Tfam* (upper panel), and mitochondrial Fusion/fission genes (lower panel) in the BAT of Control and KO mice. n = 10 per group. $p = 0.0285$ (Nrf1: Male Control vs. Female Control), $p = 0.0004$ (Tfam: Male Control vs. Female Control), $p = 0.0014$ (Tfam: Female Control vs. Female KO), $p = 0.0005$ (Mfn1: Male Control vs. Female Control), $p = 0.0016$ (Mfn2: Male Control vs. Female Control), $p = 0.0026$ (Opa1: Male Control vs. Female Control), $p = 0.0033$ (Opa1: Female Control vs. Female KO). Data are expressed as the mean ± SEM. Data were analyzed by one-way ANOVA with Tukey's post hoc test. Significance is indicated (*$p < 0.05$; **$p < 0.01$; ***$p < 0.001$). ns, not significant. Source data are provided as a Source Data file.

Supplementary Figure 3

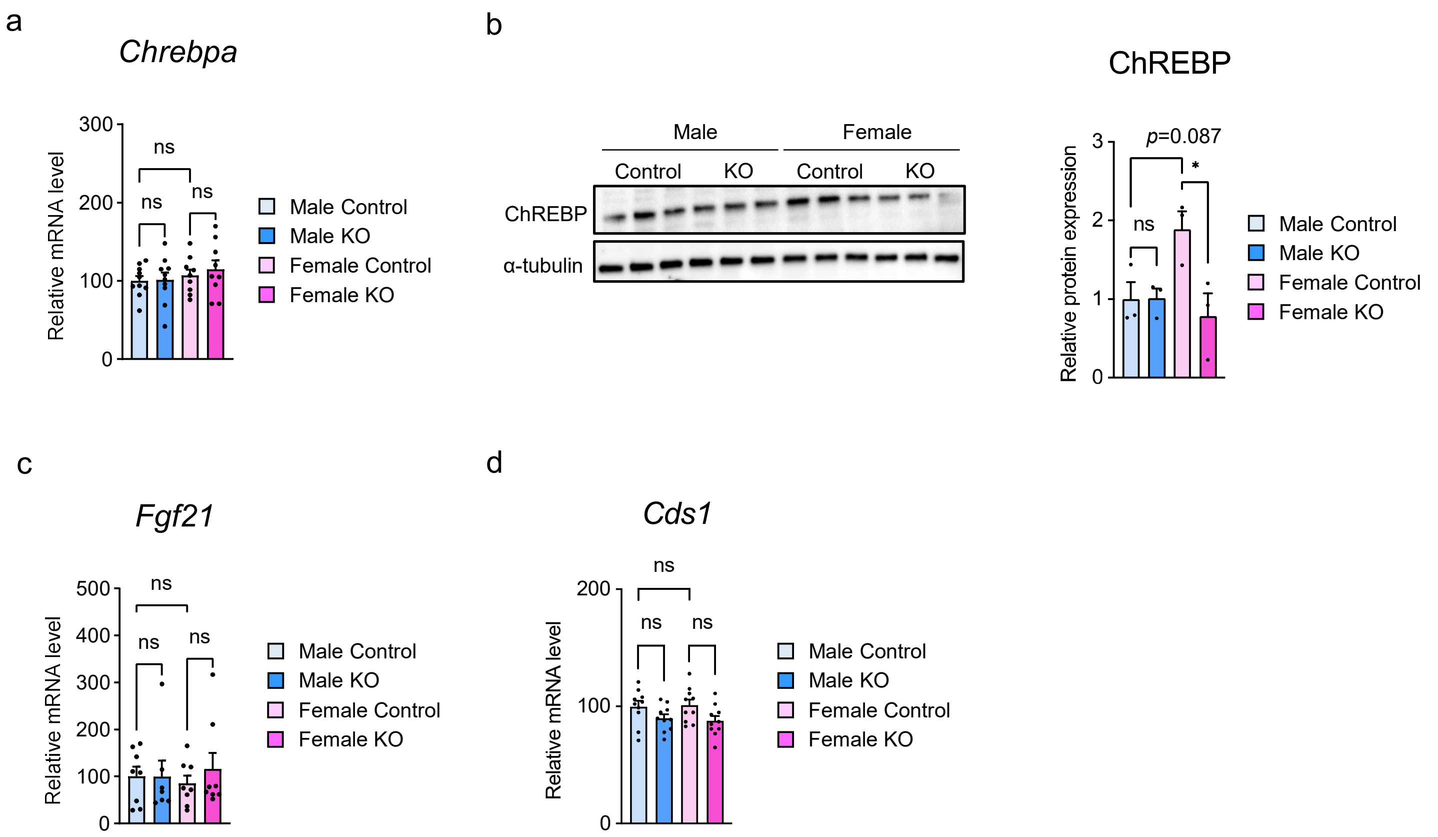

Supplementary Figure 3

PGC-1α in BAT does not affect the gene expression of *Chrebpα*, *Fgf21*, or *Cds1*

(a) Gene expression of *Chrebpα* in the BAT of Control and KO mice. n = 10 per group. **(b)** Western blots for ChREBP proteins (left) and protein levels normalized to α-tubulin (right) in the BAT of Control and KO mice. n = 3 per group. $p = 0.0873$ (Male Control vs. Female Control), $p = 0.0334$ (Female Control vs. Female KO). **(c)** *Fgf21* mRNA levels in the BAT of Control and KO mice. n = 7–8 per group. **(d)** *Cds1* mRNA levels in the BAT of Control and KO mice. n = 10 per group. Data are expressed as the mean ± SEM. Data were analyzed by one-way ANOVA with Tukey's post hoc test. Significance is indicated (*$p < 0.05$). ns, not significant. Source data are provided as a Source Data file.

Supplementary Figure 4

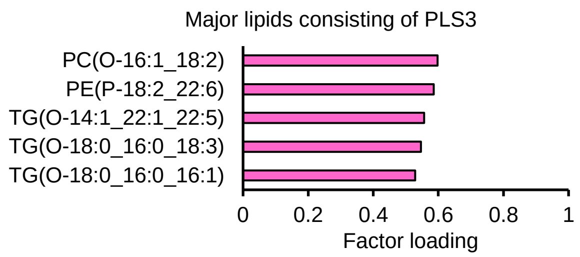

Supplementary Figure 4

Phospholipids with C > 17 fatty acids are more abundant in female mice than in male mice

Top 5 lipids contributing to PLS3 loading

Supplementary Figure 5

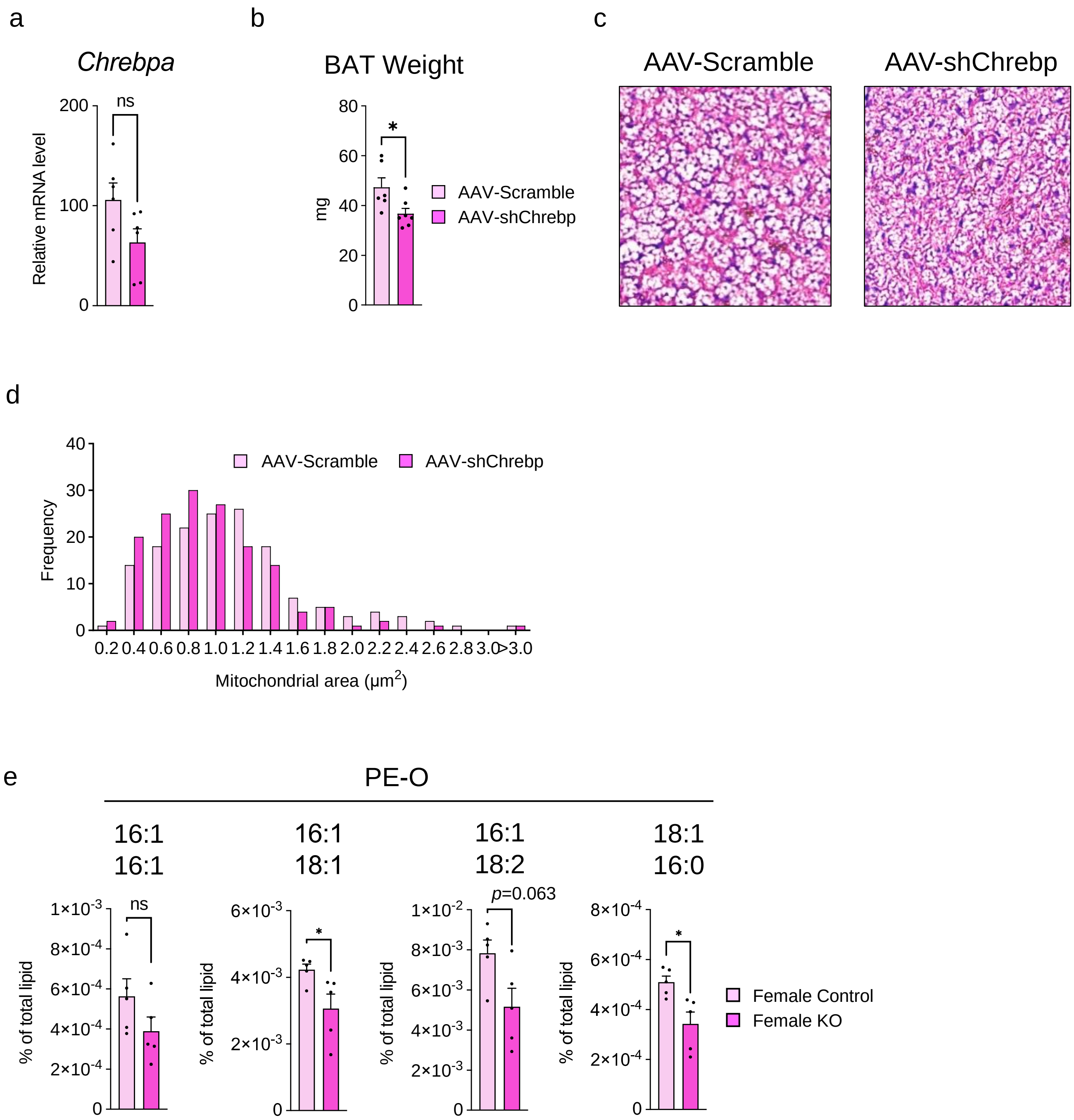

Supplementary Figure 5

Ether-linked PEs are reduced in female PGC-1α KO mice

(a) Gene expression of *Chrebpα* in female BAT injected with AAV-shScramble or AAV-shChrebp. n = 6 per group. **(b)** BAT weight. n = 6–7 per group. $p = 0.0278$. **(c)** Representative 20× Hematoxylin and eosin staining of BAT. **(d)** Histograms showing the distribution frequency (%) of mitochondrial section areas (0–3 μm^2). **(e)** Percentage of ether-linked PEs in total lipids in female Control and PGC-1α KO mice. n = 5 per group. $p = 0.0740$ (PE-O 16:1/16:1), $p = 0.0370$ (PE-O 16:1/18:1), $p = 0.0633$ (PE-O 16:1/18:2), $p = 0.0192$ (PE-O 18:1/16:0). Data are expressed as the mean ± SEM. Data were analyzed using unpaired two-sided t-test **(a, b)** and paired one-sided t-test **(e)**. Significance is indicated ($*p < 0.05$). Source data are provided as a Source Data file.

Supplementary Figure 6

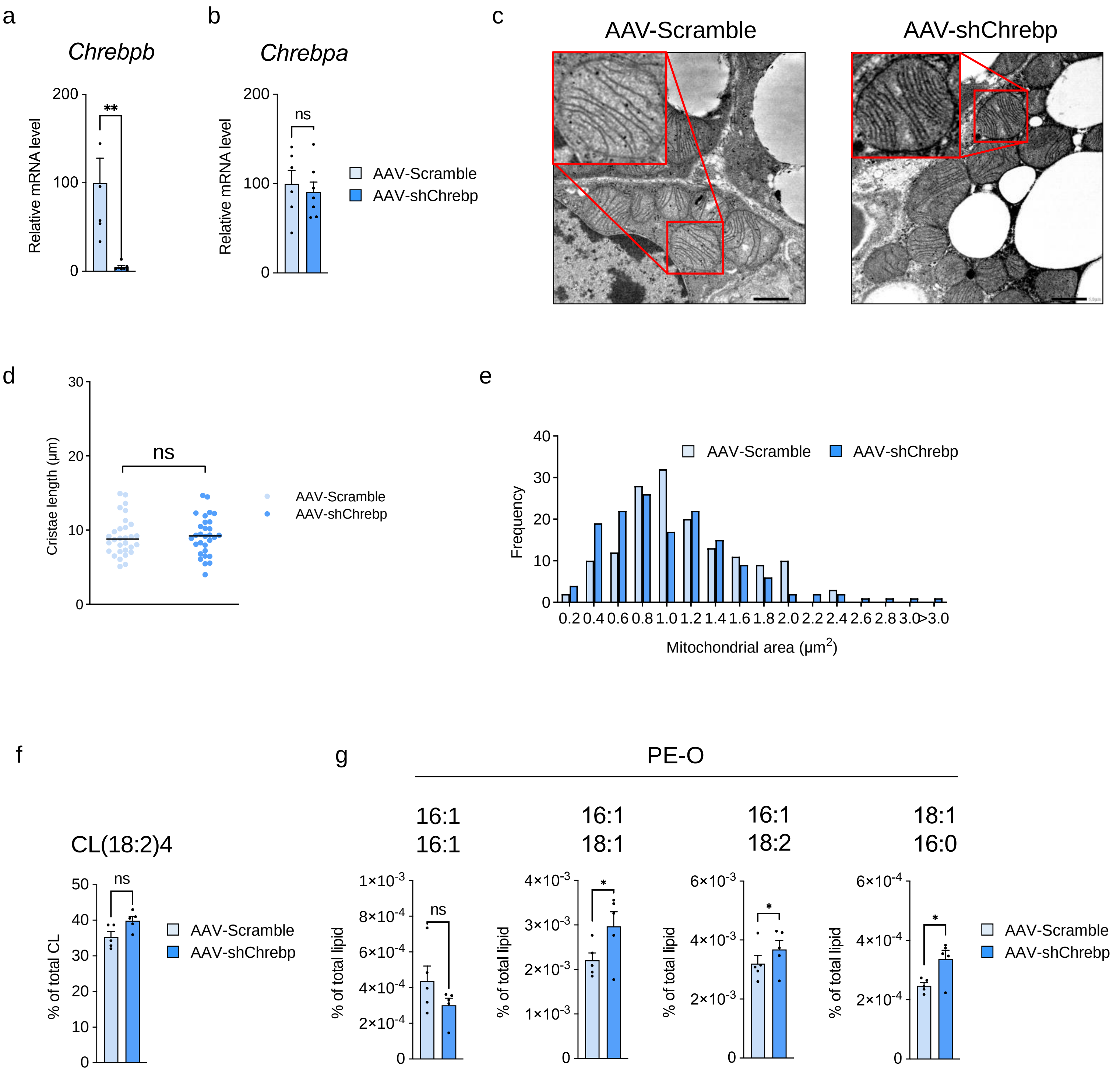

Supplementary Figure 6

ChREBPβ in male BAT is not involved in the regulation of mitochondrial morphology

(a)(b) Gene expression of *Chrebpβ* **(a)** and *Chrebpα* **(b)** in male BAT injected with AAV-shScramble or AAV-shChrebp. n = 6–7 per group. $p = 0.0036$ for **(a)**. **(c)** Representative electron micrographs of mitochondria from the BAT of AAV-Scramble and AAV-shChrebp mice. Scale bar = 1 μm. **(d)** Total cristae length per mitochondrion. **(e)** Histograms showing the distribution frequency (%) of mitochondrial section areas (0–3 μm^2). **(f)** Percentage of $CL(18:2)_4$ in total CL. n = 5 per group. **(g)** Percentage of ether-linked PEs in total lipids. n = 5 per group. Data are expressed as the mean ± SEM. Data were analyzed using unpaired two-sided *t*-test **(a, b, d)** and paired one-sided *t*-test **(f, g)**. $p = 0.0740$ (PE-O 16:1/16:1), $p = 0.0370$ (PE-O 16:1/18:1), $p = 0.0442$ (PE-O 16:1/18:2), $p = 0.0192$ (PE-O 18:1/16:0). Significance is indicated (*$p < 0.05$; **$p < 0.01$). Source data are provided as a Source Data file.

Supplementary Figure 7

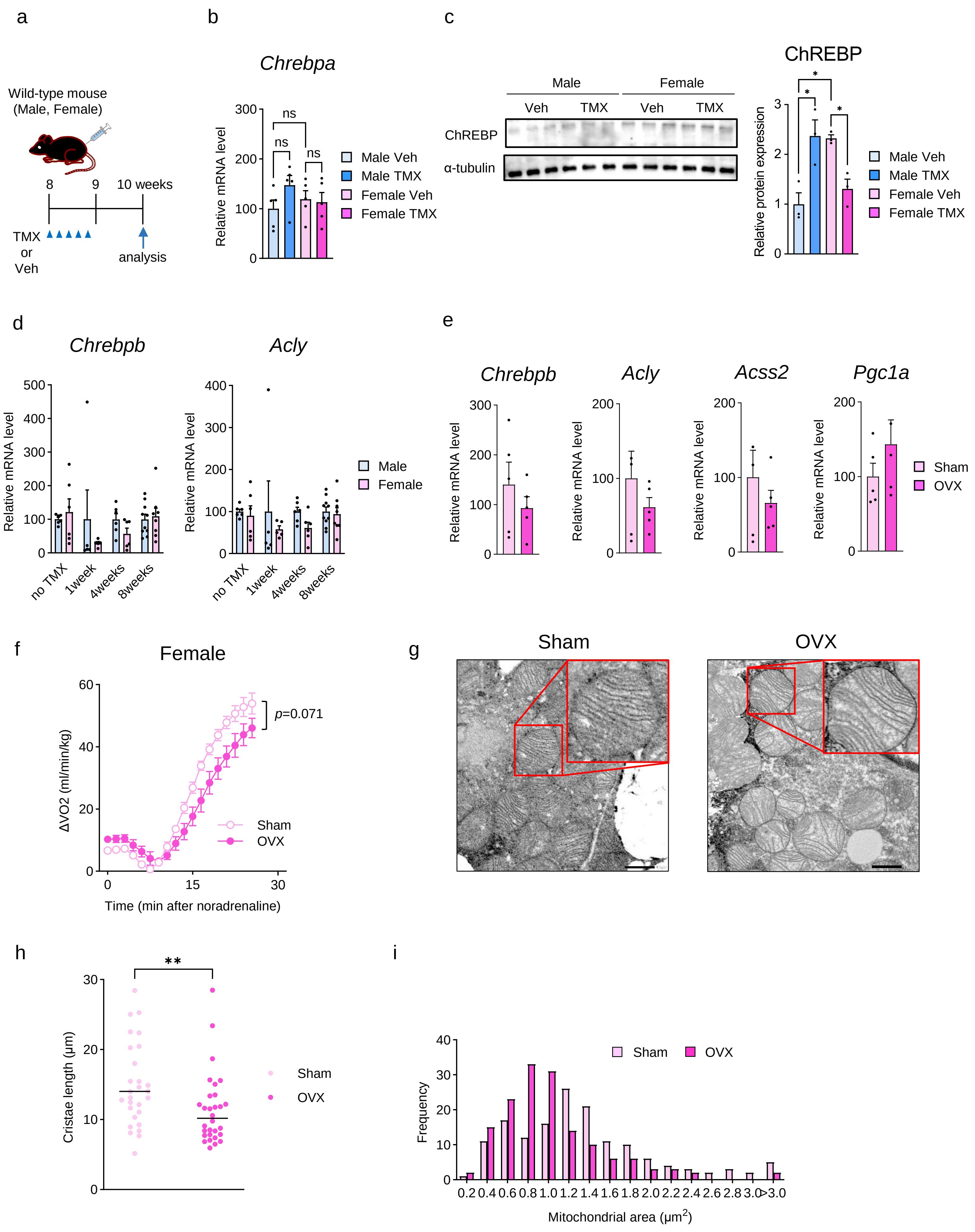

Supplementary Figure 7

Effects of ovariectomy on BAT gene expression, VO_2 after NE administration, and BAT mitochondrial morphology in female mice

(a) Experimental protocols of tamoxifen (TMX) or vehicle (Veh) treated mice. **(b)** Gene expression of *Chrebpα* in the BAT of Veh- and TMX-treated mice. n = 5 per group. **(c)** Western blots for ChREBP proteins (left) and protein levels normalized to α-tubulin (right) in the BAT of Veh- and TMX-treated mice. n = 3 per group. $p = 0.0105$ (Male Veh vs. Male TMX), $p = 0.0128$ (Male Veh vs. Female Veh), $p = 0.0491$ (Female Veh vs. Female TMX). **(d)** Gene expression of *Chrebpβ* and *Acly* in the BAT of male and female mice after 1, 4, and 8 weeks of TMX treatment. n = 5–10 per group. Note that TMX was also used to induce PGC-1α knockout, but the analysis of knockout mice was performed at least 8 weeks after TMX administration, while *Chrebpβ* and Acly gene expression in Female Control mice recovered to approximately the same level as that before TMX administration. **(e)** BAT gene expression in sham-operated (Sham) and ovariectomized (OVX) mice. n = 5 per group. **(f)** Oxygen consumption (VO_2) recordings in response to NE in Sham and OVX mice. n = 6 per group. **(g)** Representative electron micrographs of mitochondria from the BAT of Sham and OVX mice. Scale bar = 1 μm. **(h)** Total cristae length per mitochondrion. $p = 0.0079$. **(i)** Histograms showing the distribution frequency (%) of the mitochondrial section areas (0–3 μm^2).

Data are expressed as the mean ± SEM. Data were analyzed by one-way ANOVA with Tukey's post hoc test **(b, c)**, unpaired two-sided t-test **(d, e, h)**, and two-way repeated measures ANOVA **(f)**. Significance is indicated (*$p < 0.05$; **$p < 0.01$). ns, not significant. Source data are provided as a Source Data file.

Supplementary Figure 8

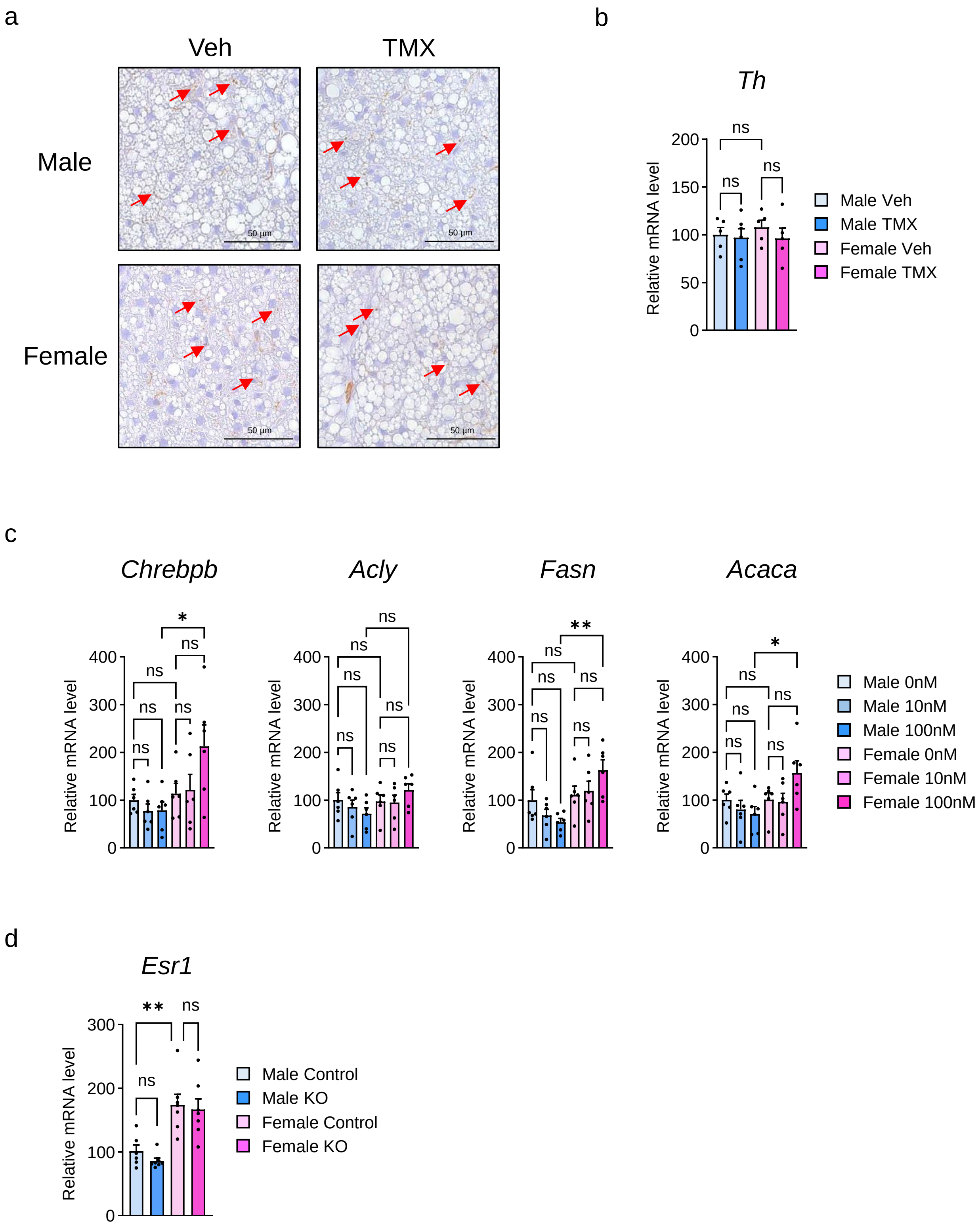

Supplementary Figure 8

TMX administration affects neither male nor female BAT sympathetic signaling

(a)(b) Tyrosine hydroxylase immunolabeling **(a)** and mRNA levels in BAT **(b)**. n= 5-6 per group. **(c)** Gene expression in BAT explants from wild-type male and female mice treated with 17β-estradiol (E2, 0nM, 10nM, 100 nM, 24 hours). n = 6 per group. $p = 0.0138$ (Chrebpβ), $p = 0.0020$ (Fasn), $p = 0.0223$ (Acaca) for Male 100nM vs. Female 100nM. **(d)** *Esr1* mRNA levels in BAT of Control and KO mice. n = 6–7 per group. $p = 0.0050$ for Male Control vs. Female Control. Data are expressed as the mean ± SEM. Data were analyzed using one-way ANOVA with Tukey's post hoc test **(b, c, d)**. Significance is indicated ($*p < 0.05$; $**p < 0.01$). ns, not significant. Source data are provided as a Source Data file.

Table S1. Mouse primers used in this study.

Gene	Forward primer(5'-3')	Reverse primer(5'-3')
Pgc1a	AGCCGTGACCACTGACAACGAG	GCTGCATGGTTCTGAGTGCTAAG
Ucp1	CGCTGGACACTGCCAAAGT	GGTGGTGATGGTCCCTAGGA
Adcy3	AGGATGAGCTGGAAGGGATG	AGGTAGAGGAAGACGTTGGC
Pgc1b	AGTCAGCGGCCTTGTGTCAA	ACTCTGGGACAGGGCAGCA
Nrf1	ACATTGGCTGATGCTTCAGAA	TGCGTCGTCTGGATGGTCAT
Tfam	TCTATCAGTCTTGTCTGTATTCCGAAGT	TGGATAGCTACCCATGCTGGA
Mfn1	AGGGGACCGATGGAGATAAAG	AAGAGGGCACATTTTGCTTTG
Mfn2	ACGTCAAAGGGTACCTGTCCA	CAATCCCAGATGGCAGAACTT
Opa1	TCACCTCTGCGTTTATTTGAAGA	GGGTAGAACGGGAGGAAAGG
Drp1	GGGCACTTAAATTGGGCTCC	TGTATTCTGTTGGCGTGGAAC
Chrebpb	TCTGCAGATCGCGTGGAG	CTTGTCCCGGCATAGCAAC
Acly	CTCACACGGAAGCTCCATAA	ACGCCCTCATAGACACCATC
Acss2	GCTTCTTTCCCATTCTTCGGT	CCCGGACTCATTCAGGATTG
Fasn	CCTGGATAGCATTCCGAACCT	AGCACATCTCGAAGGCTACACA
Acaca	GGAGATGTACGCTGACCGAGAA	ACCCGACGCATGGTTTTCA
Elovl6	TCAGCAAAGCACCCGAAC	AGCGACCATGTCTTTGTAGGAG
Chrebpa	CGACACTCACCCACCTCTTC	TTGTTCAGCCGGATCTTGTC
Fgf21	CCTCTAGGTTTCTTTGCCAACAG	AAGCTGCAGGCCTCAGGAT
Th	CCTTTGACCCAGACACAGCA	ATACGAGAGGCATAGTTCCTGAG
Esr1	CTTGGAAGGCCGAAATGAAATG	GGCAGGGCTATTCTTCTTAGTG
